# The P72R Polymorphism in R248Q/W p53 Mutants Modifies the Mutant Effect on Epithelial to Mesenchymal Transition Phenotype and Cell Invasion via CXCL1 Expression

**DOI:** 10.3390/ijms21218025

**Published:** 2020-10-28

**Authors:** Cristabelle De Souza, Jill A. Madden, Dennis Minn, Vigneshwari Easwar Kumar, Dennis J. Montoya, Roshni Nambiar, Zheng Zhu, Wen-Wu Xiao, Neeki Tahmassebi, Harikumara Kathi, Nina Nelson, Anthony N. Karnezis, Jeremy Chien

**Affiliations:** 1 Department of Biochemistry and Molecular Medicine, University of California Davis Medical Center, Sacramento, CA 95817, USA; cmdesouza@ucdavis.edu (C.D.S.); veaswarkumar@ucdavis.edu (V.E.K.); djmontoya@ucdavis.edu (D.J.M.); rnambiar@ucdavis.edu (R.N.); zegzhu@ucdavis.edu (Z.Z.); wxiao@UCDAVIS.EDU (W.-W.X.); natahmassebi@ucdavis.edu (N.T.); hrkathi@ucdavis.edu (H.K.); nrnelson@ucdavis.edu (N.N.); 2 University of New Mexico School of Medicine, Biomedical Sciences Graduate Program, Albuquerque, NM 87106, USA; 3 The Manton Center for Orphan Disease Research and The Division of Genetics and Genomics, Boston Children’s Hospital, Boston, MA 02115, USA; jill.madden@childrens.harvard.edu; 4 College of Information and Computer Sciences, University of Massachusetts, Amherst, MA 01003, USA; dminn@umass.edu; 5 Department of Biology, California State University, Channel Islands, CA 93012, USA; 6 Department of Pathology and Laboratory Medicine, UC Davis Medical Center, Sacramento, CA 95817, USA; ankarnezis@ucdavis.edu; 7 Department of Obstetrics and Gynecology, University of California Davis Medical Center, Sacramento, CA 95817, USA

**Keywords:** ovarian cancer, mutant p53, P72R polymorphism, CXCL1, tumor invasion

## Abstract

High-grade serous carcinoma (HGSC), the most lethal subtype of epithelial ovarian cancer (EOC), is characterized by widespread *TP53* mutations (>90%), most of which are missense mutations (>70%). The objective of this study was to investigate differential transcriptional targets affected by a common germline P72R SNP (*rs1042522*) in two p53 hotspot mutants, R248Q and R248W, and identify the mechanism through which the P72R SNP affects the neomorphic properties of these mutants. Using isogenic cell line models, transcriptomic analysis, xenografts, and patient data, we found that the P72R SNP modifies the effect of p53 hotspot mutants on cellular morphology and invasion properties. Most importantly, RNA sequencing studies identified CXCL1 a critical factor that is differentially affected by P72R SNP in R248Q and R248W mutants and is responsible for differences in cellular morphology and functional properties observed in these p53 mutants. We show that the mutants with the P72 SNP promote a reversion of the EMT phenotype to epithelial characteristics, whereas its R72 counterpart promotes a mesenchymal transition via the chemokine CXCL1. These studies reveal a new role of the P72R SNP in modulating the neomorphic properties of p53 mutants via CXCL1, which has significant implications for tumor invasion and metastasis.

## 1. Introduction

Ovarian cancer is the second most common and most lethal gynecological malignancy in the United States, with a 5-year survival rate of less than 50% [1]. Some of the challenges contributing to these high mortality rates are the lack of effective screening modalities, resulting in advanced stages at diagnosis, and acquired resistance to treatment leading to a higher likelihood of recurrences and eventual treatment failure and death [2,3]. There are several genetic and epigenetic determinants regulating ovarian carcinogenesis. High-grade serous carcinoma (HGSC), the most common and lethal ovarian carcinoma, is characterized by near universal mutations in *TP53* [4]. During ovarian tumorigenesis and progression, transformed cells are thought to undergo a phenotypic change called the epithelial-to-mesenchymal transition (EMT) ultimately leading to metastasis throughout the peritoneal cavity, the omentum, and even to the parenchyma of the liver or lung [5,6].

The EMT is a cellular phenotypic change that cells undergo during cancer progression. EMT is characterized by a downregulation of epithelial cell markers like E-cadherin, an upregulation of mesenchymal cell markers like N-cadherin, altered cell polarity, reorganization of cellular cytoskeleton, resistance to anoikis [7,8,9] and the dysregulation of multiple signaling pathways like the transforming growth factor (TGF)-β, Wnt, and Notch signaling pathways, all of which play key roles in oncogenesis. Tumor Associated Macrophages (TAMs) are activated macrophages found in the tumor microenvironment, which secrete a wide variety of chemokine factors that play an integral role in tumor metastasis and invasion [10,11,12].

p53, which is encoded by the *TP53* gene, is a tumor suppressive transcription factor and a master regulator of multiple biological pathways [13]. This gene is mutated in most human cancers (>50%), R248Q/W p53 mutants and a large majority of these mutations are missense mutations (70%) [14,15,16,17]. Most of the missense mutations found in *TP53* are between exons 5 and 8 in the DNA binding domain of the protein and those mutations seen at a high frequency are called hotspot mutations [18,19]. In addition to somatic mutations in the DNA binding domain of the p53 protein, inherited germline polymorphisms also exist, which can be exonic or intronic [20]. The P72R polymorphism is an evolutionarily conserved, proportionally distributed SNP found in the proline-rich domain of the p53 protein. This SNP, located in exon 4 of the *TP53* gene, either encodes for a proline (CCC) or an arginine (CGC). The P72R SNP has been shown to regulate multiple functions of p53 including cell cycle progression, apoptosis, and tumor cell invasion (De Souza, Karnezis, Chien et al., manuscript under revision) [21,22,23,24,25,26,27,28,29]. The Murphy group has reported that the R72 SNP can regulate the transcriptional activities of p53 hotspot mutants R175H and R273H to regulate tumor cell metabolism and invasion via PGC-1α [30]. However, it is unclear whether additional mechanisms exist by which the P72R SNP regulates mutant p53-mediated tumor cell invasion and whether all hotspot mutants are regulated similarly by the SNP.

The *CXC* Chemokine family consisting of *CXCL1*, *CXCL2*, *CXCL3*, *CXCL5*, *CXCL6*, *CXCL7* and *CXCL8*, are key mediators of angiogenesis and metastasis signaling via their receptors *CXCR1* and *CXCR2* in humans [31,32]. *CXCL1*, also known as *GRO-1*, is also known for its significant role in regulating the cross-talk between cancer cells and the tumor microenvironment (TME) [33]. Mutant p53 can regulate the intratumoral immune cell landscape via regulating multiple players of the secreted *CXC* Chemokine family, including *CXCL1*, and regulate VEGF signaling, angiogenesis, cell migration, and tissue inflammation [34,35,36,37]. One important study demonstrated that mutant p53 specifically exerts some gain-of-function activities by directly transactivating the *CXCL1* promoter [38].

Our studies aimed to test whether (1) R248Q and R248W p53 hotspot mutants promote invasion via CXCL1, (2) if these hotspot p53 mutants directly activate the *CXCL1* transcription, and (3) if the P72R SNP modulates this phenotype.

## 2. Results

### 2.1. The P72R SNP alters the Morphology of R248Q/W p53 Mutants

**Figure 1:**
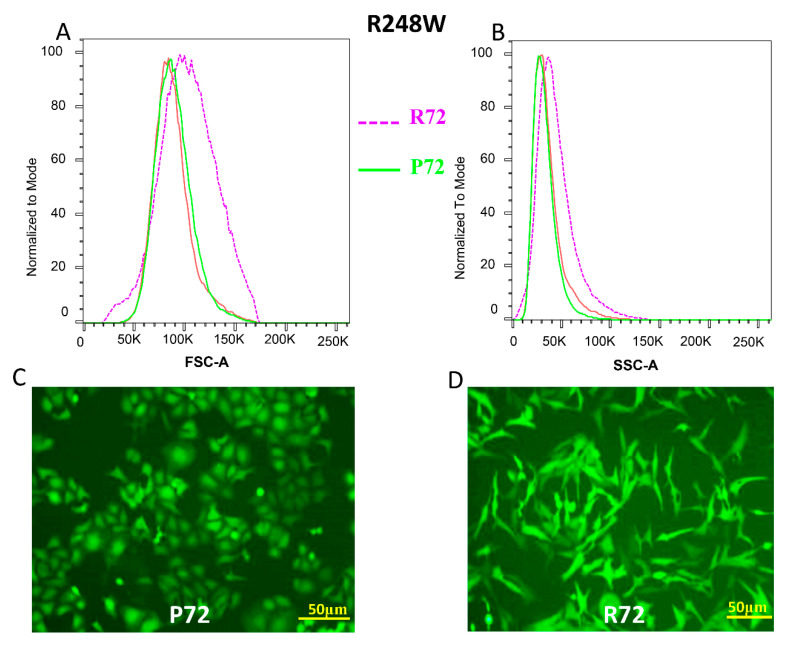

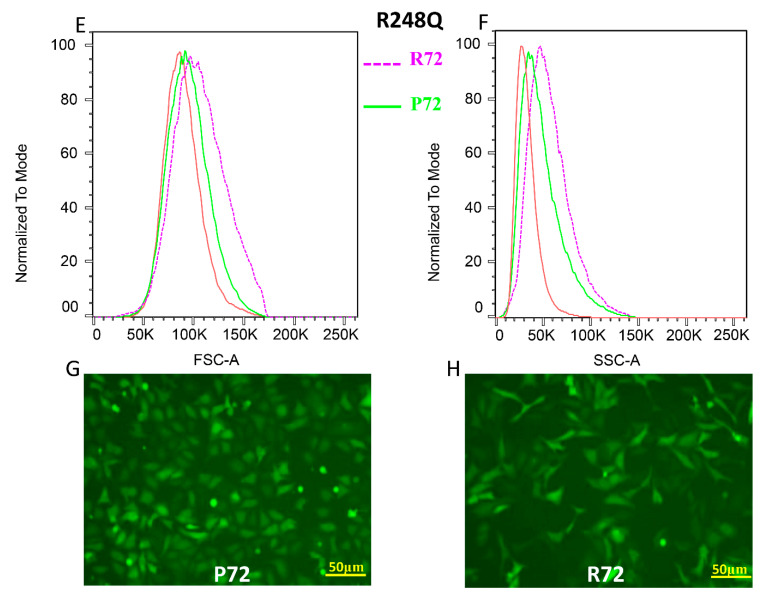
The P72R SNP alters the morphology of p53 mutants (**A**,**B**) Flow cytometry analysis showing the Forward Scatter (FSC) and Side Scatter (SSC) of p53 mutant R248W with the P72 SNP (green) and p53 mutant R248W with the R72 (purple). SKOV3 parental cells (Red) were used as unstained control for gating. (**C**,**D**) Fluorescent microscopy images showing the morphological changes observed in p53 mutant R248W with the P72 SNP (left) and p53 mutant R248W with the R72 (right). (**E**,**F**) Flow cytometry analysis showing the Forward Scatter (FSC) and Side Scatter (SSC) of p53 mutant R248Q with the P72 SNP (green) and p53 mutant R248Q with the R72 (purple). SKOV3 parental cells (Red) were used as unstained control for gating. (**G**,**H**) Fluorescent microscopy images showing the morphological changes observed in p53 mutant R248Q with the P72 SNP (left) and p53 mutant R248W with the R72 (right). In 1C, D, G and H, transduced pools were used. FSC Chi-Squared T(X) for R248W + R72 compared to R248W + P72 = 689 and SSC Chi-Squared T(X) = 526. FSC Chi-Squared T(X) for R248Q + R72 compared to R248Q + R72 = 485 and SSC Chi-Squared T(X) = 252.

Analysis of somatic mutations in TP53 in HGSC from the Cancer Genome Atlas Pan Cancer studies indicate that R273, R248, and R175 are the most frequently mutated “hotspots” in ovarian cancer (Supplementary Figure S1). Given that prior studies have already reported the modifying effect of P72R SNP on cell invasion by R175H and R273H p53 mutants [30], we focused on the remaining hotspot mutants (R248Q/W) in this study. Using an isogenic ovarian cancer cell line SKOV3 that lacks p53 expression, we generated two hotspot missense mutations R248W and R248Q, each with a respective P72 or R72 polymorphism. We observed that mutant pairs R248W P72 and R248W R72 behaved differently in terms of morphological characteristics (Figure 1C,D). The same was observed between mutants R248Q P72 and R248Q R72 (Figure 1G,H). The mutant cells with the P72 SNP retained epithelial cellular characteristics in both p53 mutants R248W and R248Q (Figure 1C,G). The R248W R72 and R248Q R72 mutants consistently displayed mesenchymal cellular characteristics (Figure 1D,H). We tried to quantitatively determine the altered cellular structural profiles within each mutant pair. Using flow cytometry analyses, we found the differential Forward Scatter (FSC) and Side Scatter (SSC) profiles for the mutant populations respectively. FSC analyses revealed that the population of cells with R248W + R72 was significantly larger (Chi-Squared T(X) = 689) when compared to the population of cells with R248W + P72 mutants. Following a similar trend SSC analyses revealed that the population of cells with R248W + R72 was significantly more complex/granular (Chi-Squared T(X) = 526) when compared to the population of cells with R248W + P72 mutants (Figure 1A–D, Supplementary Figure S2C). We also observed a similar trend in the R248Q mutant populations. FSC analyses in the two cellular populations revealed that R248Q R72 was significantly larger (Chi-Squared T(X) = 485) when compared to the population of cells with R248Q P72 mutants. 

Similar observations were made with regards to the SSC profiles where the population of cells with R248Q R72 was significantly more complex/granular (Chi-Squared T(X) = 252) when compared to the population of cells with R248Q P72 mutants (Figure 1E–H, Supplementary Figure S2D). This effect was not observed in cell populations with wild type p53 with the P72 or R72 SNP (FSC Chi-Squared T(X) = 0.83, SSC Chi-Squared T(X)- = 1.93) where the difference between the two populations (WT-R72 vs. WT-P72) was not significant (Supplementary Figure S2A,B).

### 2.2. CXCL1 is Significantly Overexpressed in p53 Mutants with the R72 SNP

**Figure 2:**
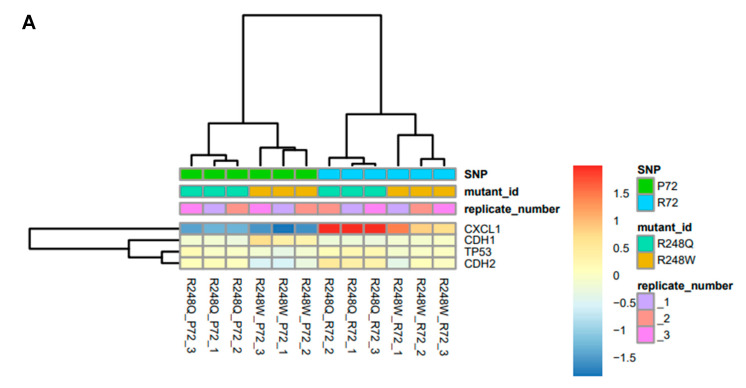

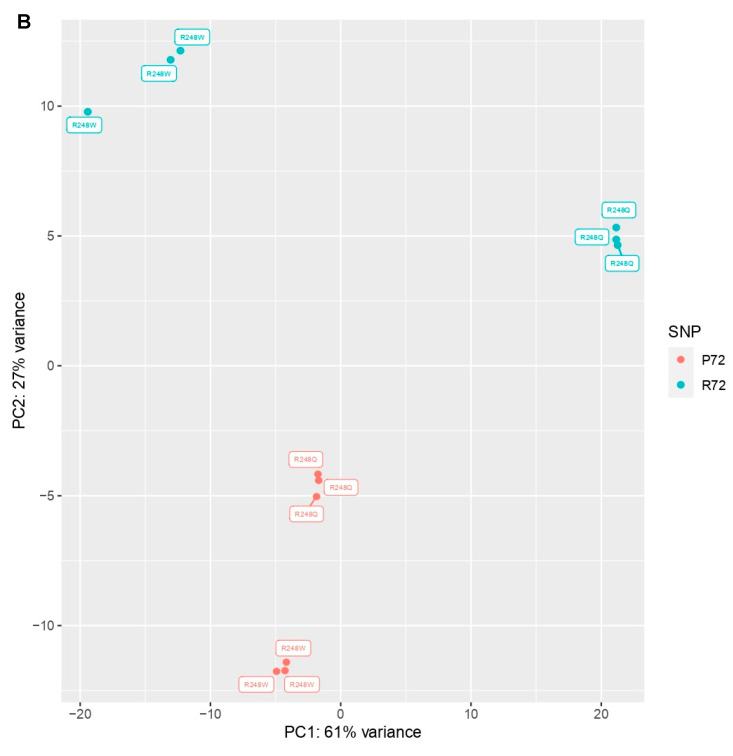

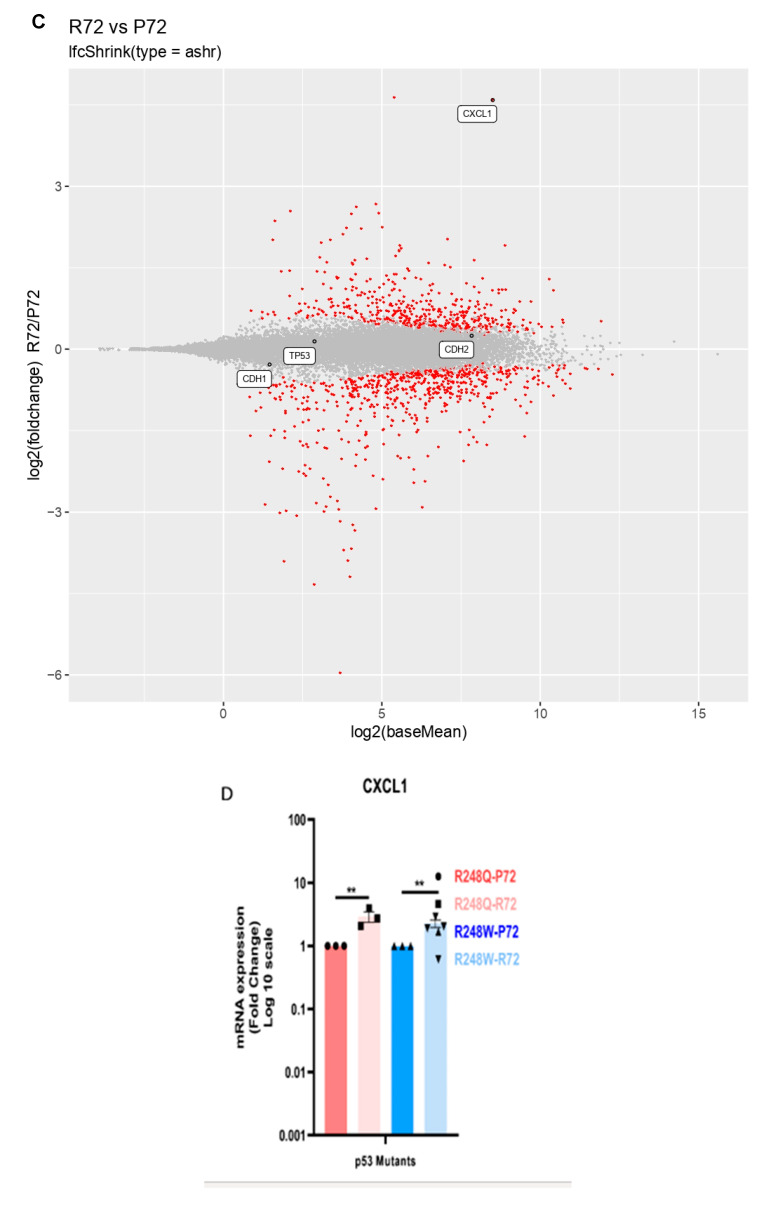

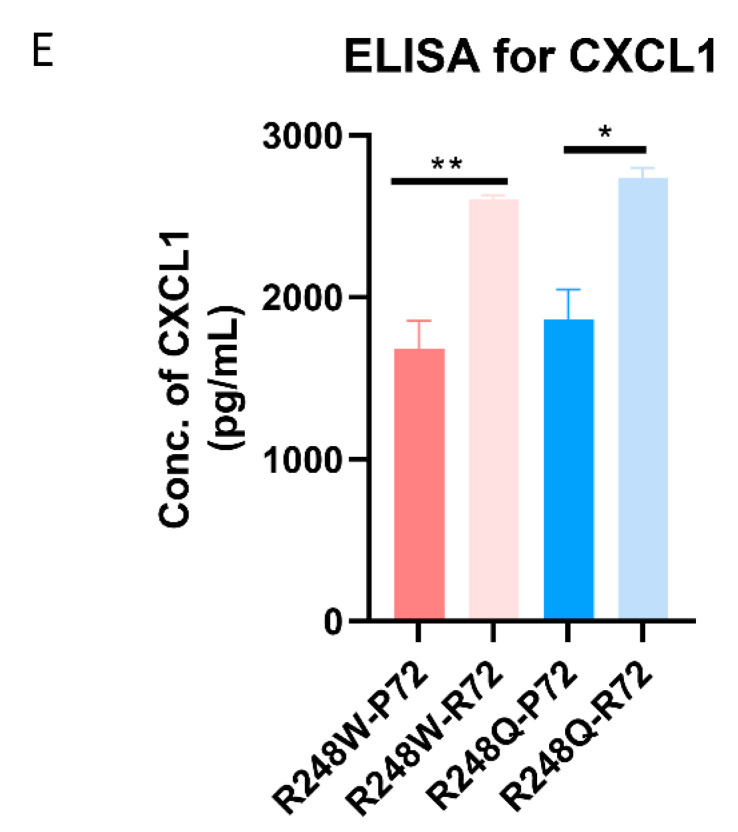
CXCL1 is significantly overexpressed in p53 mutants with the R72 SNP (**A**) Heat map showing gene expression profiles of mutants R248W and R248Q with the P72 or R72 SNP. Transcripts for CXCL1, CDH1, CDH2 and TP53 are shown. Experiment consists of three independent biological and technical replicates. (**B**) Principal Component Analysis (PCA) indicating percent variance between the respective mutant pairs. (**C**) MA plots showing CXCL1 significantly overexpressed relative to CDH1, CDH2 and TP53 across both p53 mutant pairs (R72 group *n* = 6, P72 group *n* = 6). (**D**) mRNA expression profiles of each pair of mutant shows significant overexpression of R248Q P72 compared to R248Q R72 and R248W P72 compared to R248W R72 using RT-qPCR. (**E**) Sandwich ELISA assay used to determine concentration of CXCL1 in the cellular media supernatant for each mutant pair. All data shown as mean ± SEM. The statistical analysis was performed using two-tailed Student’s t-test: * *p* ≤ 0.05, ** *p* ≤ 0.01,

We then sought to understand the mechanism underlying the differential cellular morphological characteristics being regulated by the P72R polymorphism in mutant p53. We performed 3′-tag RNA sequencing and carried out pair-wise analysis to reveal altered transcript levels between the mutants and their respective SNPs. The heat map obtained shows that both mutants R248W R72 as well as R248Q R72 have significantly (*p* < 0.05) upregulated transcripts of CXCL1 (Figure 2A, Supplementary Figure S3A). The P72 SNP in both R248W and R248Q do not express CXCL1 transcripts at that level (Figure 2A,C, Supplementary Figure S3A,B,D). Even though differences exist in conventional markers of the EMT phenotype like CDH1 (E-cadherins) and CDH2 (N-cadherins) the differential expression observed in CXCL1 is the highest transcript upregulated in the mutant with R72 (Figure 2A,C). Principal Component Analysis (PCA) performed on the samples shows a strong correlation between the different replicates and emphasizes low variations with strong patterns, indicating that there is a consistent increase in CXCL1 transcript levels across all replicates of the samples (Figure 2B). To further validate our data, we used pair-wise analysis of both the P72 mutants versus both the R72 mutants (*n* = 6 per group, 12 samples in total). The MA plot shows that CXCL1 is consistently upregulated by both p53 mutants with the R72 SNP’s irrespective of mutation status (Figure 2C, Supplementary Figure S3A). This suggests that the R72 SNP can regulate CXCL1 irrespective of the hotspot mutation status (R248W or R248Q) but mutation-specific levels of expression may differ based on cell or cancer type. We also performed quantitative RT-PCR to check for RNA expression profiles of the mutants with the SNP. Here, we show that even though the p53 expression levels between all mutants is similar, the CXCL1 mRNA levels are significantly upregulated by the R72 SNP p53 mutants (Figure 2D, Supplementary Figure S3C). We also assessed the expression levels of conventional markers of EMT and found that in both the respective pairs of mutants, CDH1 expression levels did not vary, whereas CHD2 levels significantly (*p* < 0.05) differed such that mutants R248W R72 and R248Q R72 had significantly upregulated CDH2 levels, which is consistent with the phenotypic mesenchymal cellular behavior observed (Supplementary Figure S3C). 

We also identified additional transcripts that were significantly upregulated by the R72 mutants and found CXCL1, CXCL2, ALDH3A1 and MAP2K6 as transcripts that were significantly upregulated by the R72 mutants (Supplementary Figure S3D). Since CXCL1 is a soluble chemokine secreted extracellularly by cells, we determined the levels of secreted CXCL1 between the mutants with the R72 and P72 using capture ELISA specific for CXCL1. Our data indicates that R248W R72 and R248Q R72 mutant cell lines have a significant over secretion of CXCL1 relative to their counterparts R248W P72 and R248Q P72 (Figure 2E).

### 2.3. The P72R SNP Alters the Invasion Profile of Mutant p53 via CXCL1

**Figure 3:**
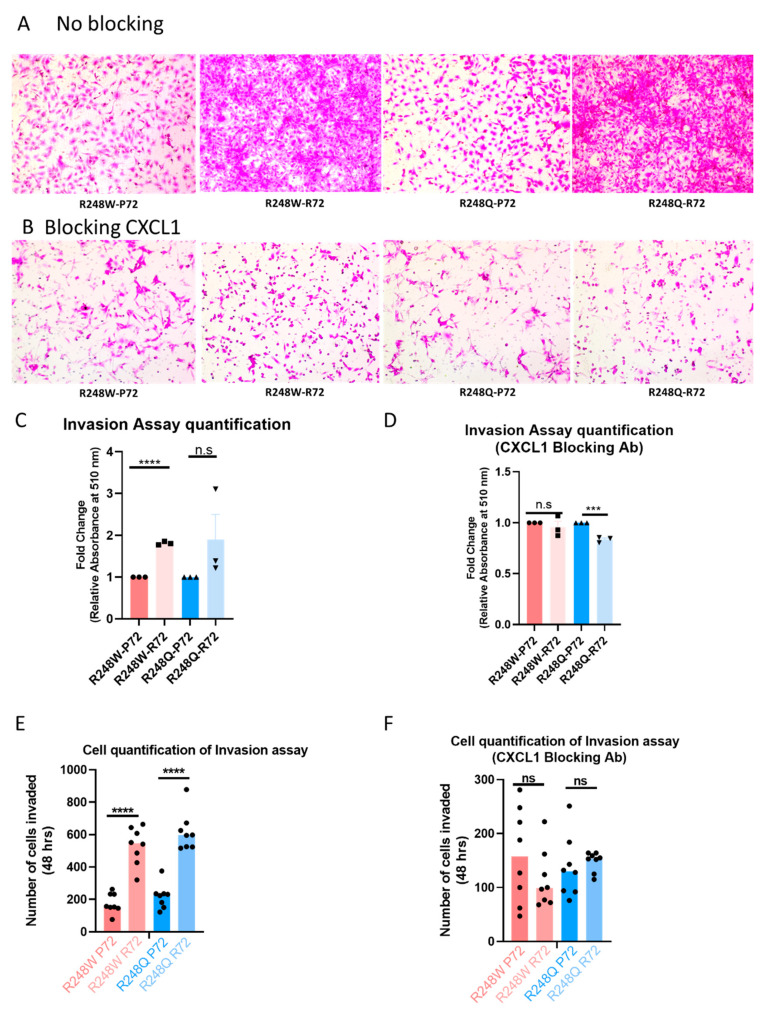
The P72R SNP alters the invasion profile of mutant p53 via CXCL1 (**A**) Boyden chamber transwell assay denotes invasion pattern of SKOV3 using conditioned media obtained from each of the mutants. Conditioned media was untreated with CXCL1 blocking antibody. Representative Images displayed. All images available in supplementary figures. (**B**) Boyden chamber transwell assay denotes invasion pattern of SKOV3 using conditioned media obtained from each of the mutants. Conditioned media was treated with CXCL1 blocking/neutralizing antibody (40 ng/mL). Representative Images displayed. All images available in supplementary figures. (**C**,**D**) Quantification of cells that were invaded by fixation followed by staining with SRB dye. Absorbance was measured at 510nm. (**E**,**F**) The total number of cells that invaded in 48 hrs were quantified for both mutants in eight different relicates. All data shown as mean ± SEM. The statistical analysis was performed using two-tailed Student’s t-test: *** *p* ≤ 0.001, **** *p* ≤ 0.0001.

To functionally assess the invasion profiles of the mutants with the P72 and R72 SNPs, we performed the Boyden chamber assay with parental SKOV3 cells and used conditioned media from each of the mutant pairs as chemoattractant. Our results indicate that SKOV3 parental cells displayed a greater invasive potential when subjected to conditional media from mutants with the R72 SNP. Invasion of SKOV3 cells in response to conditional media from mutants with P72 SNP was significantly lower (Figure 3A, Supplementary Figure S4A). The total number of cells invaded was also quantified colorimetrically to demonstrate a significant difference in the invasion profile of SKOV3 with media from the mutants with the P72 SNP versus media from mutants with the R72 SNP (Figure 3C). We further demonstrate that neutralizing CXCL1 with an antibody specific to CXCL1, exhibited no significant difference in the invasive potential of SKOV3 cells with media from the R248W P72 or R248W R72 mutants. In fact, SKOV3 cells with conditional media from the R248Q R72 mutants displayed a significantly decreased invasion after blocking CXCL1 (Figure 3B,D).

### 2.4. The R72 SNP Exhibits Higher Expression in Vivol and Enhances the Transactivation of CXCL1

**Figure 4:**
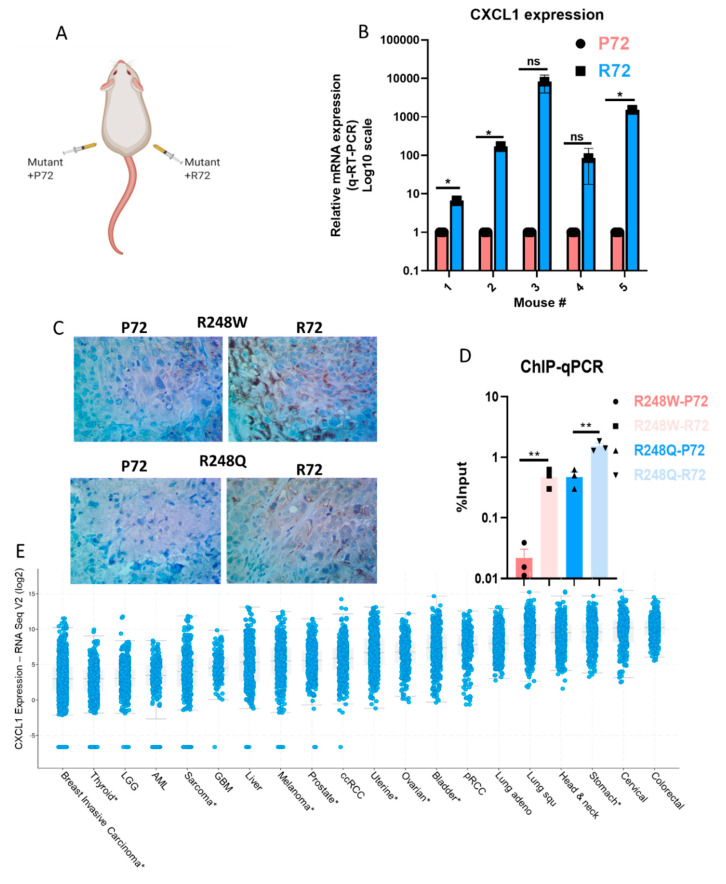
The R72 SNP exhibits higher expression of CXCL1 in animal tissue via enhanced transactivation of *CXCL1* (**A**) Schematic describing design of subcutaneous tumor injection in mouse model. (**B**) Gene expression data from RT-qPCR obtained from mouse tumors with the R248W-R72 versus mouse tumors from the R248W-P72 tumors. *X*-axis denotes the independent tumors with P72 (*n* = 5) and R72 (*n* = 5). Data are displayed in the Log10 scale. (**C**) CXCL1 expression by immunohistochemistry in tumor xenograft produced by SKOV3 cells transduced with R248W or R248Q mutants with P72 or R72 SNP. (**D**) Chromatin-Immunoprecipitation (ChIP)-qPCR data showing the enhanced binding of p53 mutants with the R72 SNP to the promoter of CXCL1 as a measure of input percentage. (**E**) CXCL1 expression in tumor samples from the Cancer Genome Atlas pan-cancer studies was obtained from the *Cbioportal*. CXCL1 expression is grouped by tumor type and ordered by median expression. Only data sets with at least 200 tumor samples are included in the plot. Asterisk denotes 2018 pan-cancer data sets. All data shown as mean ± SEM. The statistical analysis was performed using two-tailed Student’s t-test: * *p* ≤ 0.05, ** *p* ≤ 0.01.

To corroborate our in vitro data, we used tumor tissue obtained from animal studies conducted using the mutant cell lines, the schematic of which is shown in Figure 4A. 

To validate the expression of CXCL1 transcripts from in vivo tumor tissue, we isolated tumors from mice with R248W P72 and R248W R72 (*n* = 3 for R248W and *n* = 2 for R248Q, *n* = 5 total). Quantitative RT-PCR performed on the tumor tissue revealed an overall upregulated expression of CXCL1 transcripts in the tumor tissues obtained from mutants with the R72 SNP thus indicating that the mutants with R72 SNP maintain CXCL1 expression in vivo while the mutants with the P72 SNP have a reduced CXCL1 expression (Figure 4B). To further qualitatively visualize the increased CXCL1 expression in mouse tumor tissue, we performed IHC and show that in both mutants R248W and R248Q, the R72 SNP maintains relatively higher levels of CXCL1 expression compared to their P72 counterparts (Figure 4C, Supplementary Figure S5A,B). To define the mechanism underlying mutant p53′s regulation of CXCL1, we used chromatin immunoprecipitation (ChIP) followed by quantitative PCR specific for p53 binding to the promoter of CXCL1 and found that mutants with the R72 SNP had an enriched binding to the promoters of CXCL1, thus providing evidence to suggest that both mutants R248W and R248Q with the R72 SNP significantly enhances transactivation of CXCL1 (Figure 4D). We also analyzed CXCL1 expression in tumor samples from the TCGA data sets curated in Cbioportal and show that CXCL1 expression is elevated in multiple cancer types especially in ovarian cancer (Figure 4E). We then attempted to recapitulate these findings in human tumor specimens by investigating the CXCL1 expression of ovarian cancer tumors from the TCGA database that have p53 missense mutations (*n* = 185). Of these samples, those homozygous for R72 (*n* = 88) had a CXCL1 mRNA expression higher than those homozygous for the P72 alelle (*n* = 39), but it did not reach significance (fold change = 8.25, *p* = 0.2) (Supplementary Figure S6A). Furthermore, in those samples that specifically had p53 R248 missense mutations, there also showed a trend toward a higher expression in R72 homozygous tumors (fold change = 7.8, *p* = 0.3) (Supplementary Figure S6B). Based on our data and analysis, we propose a potential mechanism through which the P72R polymorphism rs1042522 could be potentiating the invasiveness of cancer cells via mutant p53 (Graphical Abstract). In summary, we suggest that CXCL1 is an important protein that regulates tumor cell invasion and the expression patterns of CXCL1 are modulated by the missense mutation status and the inherited variation status at position 72 in the p53 protein.

## 3. Discussion

Ovarian cancers are characterized by genomic alterations leading to altered transcriptional networks. One such altered network is the EMT transcriptional network that is known to drive malignant progression. There are several known and unknown factors governing EMT in ovarian cancers, but most of their mechanisms remain elusive [39]. Prior studies have described the role of CXCL1 as an integral chemokine ligand that regulates the EMT transition by regulating the crosstalk between cancer cells and tumor microenvironment [33]. Previous studies have also described the effects of mutant p53 in regulating invasion and the role of the P72R polymorphism regulating the Gain of Function invasion in p53 mutants [30]. Using two hotspot p53 mutants–R248W and R248Q–we have demonstrated that the P72R polymorphism regulates a differential EMT phenotype in the background of mutant p53. Our results utilize quantitative flow cytometry analyses (FSC and SSC profiles) as well as fluorescence microscopy to show that mutant p53 in the background of the P72 SNP significantly differs from its R72 counterpart with regards to the morphological phenotype. These results are consistent with previous findings that demonstrate mutant p53 to regulate a mesenchymal phenotype as part of its *gain of function* property [40]. However, the novel result of our study shows that this *gain of function* property of R248W/Q mutants is dependent on the germline background of the P72R polymorphism and in part mediated by differential expression of CXCL1. Therefore, the P72R SNP appears to play a role in regulating the morphology of cells with mutant p53 where a mutant in the background of the P72 SNP would have more epithelial like characteristics and the mutant in the background of the R72 SNP would have more mesenchymal like characteristics.

The effect of the gain-of-function R248Q p53 mutant on cancer cell invasion by has been previously reported [41]. These studies showed the R248Q mutant decreases the expression of ZEB1 and N-cadherin and inhibit motility and invasion of breast and lung cancer cells. These studies however did not report the status of P72R polymorphism. Our results showing that R248Q and R248W mutants with the P72 polymorphism decrease ovarian cancer cell invasion is consistent with these prior reports. However, our results also indicate that negative effect on cancer cell invasion is dependent on P72R polymorphism because the same mutants with R72 polymorphism promote cancer cell invasion.

It should be noted that the expression of mutants with the R72 polymorphism is noticeably stronger than those with the P72 polymorphism. These results further support our general conclusion that mutants with the R72 polymorphism exhibit the *gain of function* effect. The lentiviral expression system we employed in this study also included green fluorescence protein (GFP) under a separate promoter. In concordance with the p53 expression, we observed correspondingly higher expression of GFP (Supplementary Figure S7) in cells transduced with the mutant p53 with the R72 polymorphism compared to the corresponding mutant with the P72 polymorphism. These results suggest the tolerance of high copy number for the mutant p53 with the R72 polymorphism and lower tolerance for high expression of the mutant with the P72 polymorphism. Finally, although the R72 and P72 sequence variations in the context of somatic mutations in p53 could differentially affect the stability and therefore the steady state expression of p53, our results indicating that both GFP and p53 mutant with the P72 polymorphism are expressed at lower levels than the R72 counterparts suggest copy number or gene dosage effect may be the primary contributing factor to the differences in p53 expression.

We performed global transcriptomic analyses to identify the molecular determinants regulating this phenotypic change between the p53 mutants. We found that *CXCL1* is the most highly upregulated gene by p53 mutants with the R72 SNP, but it is downregulated by p53 mutants with the *p* SNP. This is the first time that *CXCL1* has been shown to be differentially regulated by mutant p53 depending on the genotype of the P72R SNP. This effect is likely due to differential binding of mutant p53 proteins to the endogenous *CXCL1* promoter, dependent on the P72R SNP. Our studies are significant because we provide a possible mechanism through which mutant p53 with the R72 SNP could enhance transactivation of the *CXCL1* gene and promote invasion and potential metastases. The inability of mutant p53 in the background of the P72 SNP to bind to and transactivate the *CXCL1* promoter regions suggests a possible anti-invasive effect for these mutants. This differential transcriptional effect is also observed in tumor xenograft models, where tumors with p53 mutants in the R72 SNP background express *CXCL1* at higher levels than mutants with the P72 SNP.

Furthermore, we show that this differential expression of *CXCL1* has functional consequences in cellular invasion profiles which can be rescued by blocking *CXCL1*. This enhanced invasive effect further demonstrates that hotspot p53 mutants with the R72 SNP promote cell invasion and exhibit an *‘oncogenic’* effect. Based on our results, we propose a potential mechanistic model to explain how mutants with the R72 and P72 SNP can differentially regulate invasion through *CXCL1 (Graphical abstract)*. One limitation of this study is that we did not formally assess for invasion or metastasis *in vivo*. Our subcutaneous xenograft model may not be ideal to identify key steps involved in the intravasation and extravasation of tumor cells.

Our results have significant implications for understanding the mechanism of invasion of cancers driven by mutant p53. While we specifically focused on ovarian HGSC, our patient data analyses reveal that *CXCL1* is highly expressed in several human cancers, making it an attractive potential therapeutic target to prevent invasion and metastasis in many cancer types.

In conclusion, our study demonstrates that two common p53 hotspot mutants–R248Q and R248W–differentially regulate CXCL1 mRNA, protein, and tumor cell invasion. This differential regulation is dependent on the P72R SNP and most likely mediated by differential direct binding of mutant p53 to the *CXCL1* promoter. *CXCL1* is expressed in a wide range of human cancers which makes it a potential therapeutic target to inhibit invasion and metastasis.

## 4. Materials and Methods

### 4.1. Cell Lines and Cell Culture

SKOV3 cells obtained from ATCC and STR genotyped (Laragen, Culver City, CA, USA) was maintained in MCDB105 and M199 (1:1) (Sigma-Aldrich, St. Louis, MO USA) containing 5% FBS (Sigma) and 1% penicillin-streptomycin (15070063, Thermo Fisher, San Francisco, CA, USA). All mutant cell lines generated in SKOV3 were maintained in the same culture conditions as the parental cells. All experiments performed on cells that were passaged <20 times.

### 4.2. Plasmid and Lentiviral Particle Production

Plasmid pLenti-GIII-CMV-GFP-2A-Puro was purchased from abmgood. P53 mutants were generated using Site Directed Mutagenesis kit (Agilent, Santa Clara, CA, USA) with specific primers. Viral particles were produced by transient transfection of specific plasmids with psPAX2 and pMD2.G (Addgene, Watertown, MA, USA) into HEK293T cells using Lipofectamine 3000 (Life Technologies, Carlsbad, CA, USA). Media was collected 48 h after transfection and centrifuged at 2000 rpm at 4 °C for 10 min to pellet cell debris. The supernatant was filtered through a 0.45 μm low protein binding membrane (Steriflip HV/PVDF, Millipore, Burlington, MA, USA). Stable cell lines were generated by transducing equally titered virus particles (fresh particles used every time) followed by selection with 8 micrograms/mL of puromycin (A1113802, Thermo Fisher) for 810–days.

### 4.3. Antibodies

Mouse monoclonal p53 antibody (sc-98) was purchased from Santa Cruz Biotechnology (Dallas, TX, USA). Beta actin (8H10D10) from Cell Signaling Technologies (Danvers, MA, USA) was used. For secondary antibodies, horse anti-mouse IgG-HRP antibody (7076S) was purchased from Cell Signaling Technologies, goat anti-rabbit IgG-HRP antibody (sc-2030) was obtained from Santa Cruz Biotechnology. For CXCL1 neutralizing antibody, CXCL1 (R and D systems, catalog # MAB453-SP) was used. For CXCL1 immunoblotting, immunofluorescence, Gro alpha (CXCL1) antibody from Proteintech (Rosemont, IL, USA) was used (12335-1-AP). For immunofluorescence, the secondary antibody used was goat anti-rabbit Dylight645.4.4. ELISA Assay

Sandwich ELISA was performed using the Human CXCL1 ELISA kit (Catalog number: KE00133, Proteintech, Rosemont, IL, USA) and the protocol was followed as per the manufacturer’s instructions.

### 4.4. Gene Expression Analysis

RNA isolation, library preparation, sequencing and analysis was performed as previously described [42]. Quantitative reverse transcription PCR (RT-qPCR) was performed on RNA samples collected and processed as described by us previously [43]. For our RNA sequencing data analyses, we utilized the popular R package DESeq2 to produce the figures in our gene expression analysis [44]. To account for specific coefficients, we added “SNP” and “mutant ID” columns to our design table and specified these metadata in the design parameter when creating our DESeq2 data frame. We then used the package’s regularized log transformation function to scale the original count data by log2. This normalizes the count data with respect to each sample’s size and reduces variability among samples with low count data. The top 500 varied genes from our log transformed data is used in our principal component analysis while *CXCL1, CDH1, TP53,* and *CDH2* are subsetted and plotted in our heatmap. Utilizing our original DESeq2 data structure, gene expression analysis is conducted through DESeq2′s built in functions. These results are recorded and put into tables. These tables include our data for R72 vs. P72, R278Q R72 vs. R278 P72, etc. These result tables contain a gene’s base means, fold changes values, and adjusted *p* values. Adaptive shrinking or “ashr” is used to control false positives rates, primarily for genes with high fold change values but low base means. Fold changes and base means are plotted to form our MA graph. All points with adjusted *p* values under or equal to 0.05 are colored red, otherwise grey. In a separate graph, adjusted *p* values and fold changes are plotted to form our volcano plot. Genes in the graph that meet both the adjusted *p* value threshold and fold change threshold are colored red, otherwise grey. Analysis of TCGA ovarian cancer patients.

The Cancer Genome Atlas ovarian cancer (TCGA-OV) primary tumor samples with p53 missense mutations were selected through the Genomic Data Commons Data Portal [45] P72R genotype status of these samples was determined through analysis of exome reads in the Cancer Genomics Cloud environment [46]. First, Samtools Mpileup CWL1.0 app was used to compile reads, with subsequent calling by Bcftools Call app. Calls were then filtered by Bctools Filter for those samples with at least 10 reads at the P72R locus.

TCGA ovarian RNA sequencing raw counts were obtained through the ‘TCGAbiolinks’ R package “GDCdownload()” and “GDCprepare()” commands [47,48] Samples with RNAseq data available were then filtered by those with P72 (*n* = 39) or R72 (*n* = 88) homozygous calls. Ensembl gene ids were converted to gene symbols by the ‘GeoTcgaData’ R package “id_conversion()” command. Genes with fewer than 5 reads in 10% or less of samples were filtered out. Filtered raw counts were then normalized by R package ‘DESeq2′ [44]. CXCL1 normalized counts and P72R calls are available at the following link: https://osf.io/an4xr/Chromatin Immunoprecipitation (ChIP) and quantitative polymerase chain reaction (q-PCR). ChIP-qPCR was performed by the protocol previously described [49,50] with a few modifications from our group [51].

### 4.5. Mouse Studies

Xenografts mice were established by injecting animals subcutaneously with p53 mutant cells on either flank. The animal studies were performed according to the guidelines of the institutional animal care and local veterinary office and ethics committee of the University of California, Davis. Five mice per group for subcutaneous injection were included with a total of 10 mice. Mouse tumor tissue was collected, and RNA was extracted for cDNA synthesis for RT-qPCR and for Immunohistochemistry analysis.

### 4.6. Immunohistochemistry (IHC)

The tumor tissues were sectioned at 4 μm and subjected to IHC by standard procedures using the following antibodies: *CXCL1* (Proteintech 12335-1-AP) at a dilution of 1: 500.

### 4.7. Flow Cytometry

Flow cytometric analysis was carried out on cells fixed with 4% paraformaldehyde and analyzed using a LSRFortessa system (BD, San Jose, CA, USA). The FITC channel was used to visualize cells with GFP. Forward Scatter and Side Scatter were analyzed from the selected cell population using FlowJo version 8 (FlowJo, Ashland, OR, USA).

### 4.8. Invasion Assay

Transwell cell invasion assay was performed as previously described with 8 micron pore size inserts [52]. SKOV3 cells (20,000–50,000 cells/well) were seeded in low serum media into the cell inserts. Conditioned media supernatant obtained from the culture plates (24 well) of each p53 mutant cell line was supplied at the bottom of the well insert (200 μL). Cells were fixed after 4872– hr. incubation periods with 4% PFA. Sulphorhodamine B (SRB) dye was used to stain cells and 1% acetic acid was used to wash the stained cells. Dye was solubilized in Tris base solution before absorbance was read at 510 nm on a microplate reader (Molecular Devices, San Jose, CA, USA).

### 4.9. Statistics and Reproducibility

The differences in all assays were analyzed by two-tailed Student’s t-tests using GraphPad Prism 8 (GraphPad Software, San Diego, CA, USA). Statistical significance was set at *p* < 0.05. All experiments were carried out with at least three biological replicates. Results are presented as average (SEM), as indicated in the figure legends.

## Data Availability

The data underlying this article are available in the Open Science Framework repository and can be accessed at https://osf.io/an4xr/.

## Supplementary Materials

The following are available online at https://www.mdpi.com/1422-0067/21/21/8025/s1: list contents.
