# Supplementary material for: The P72R Polymorphism in R248Q/W p53 Mutants Modifies the Mutant Effect on Epithelial to Mesenchymal Transition Phenotype and Cell Invasion via CXCL1 Expression
Source: Int J Mol Sci. 2020 Oct 28;21(21):8025. doi: 10.3390/ijms21218025 (PMC7662892; doi:10.3390/ijms21218025)
Supplement: Supplementary file 1 [file ijms-21-08025-s001.pdf]

Supplementary Information:

Figure S1

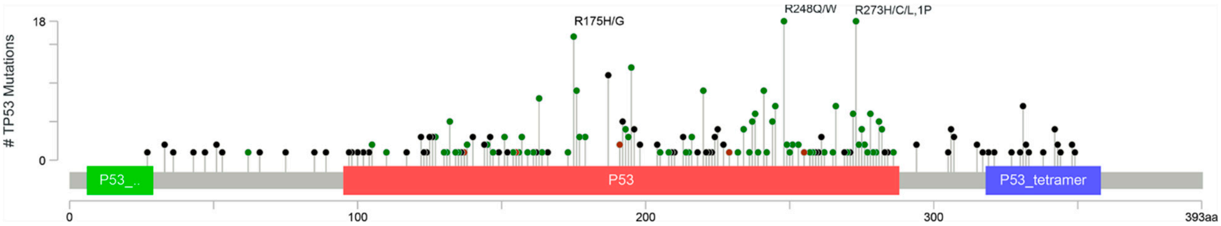

Figure S1: Hotspot mutations in TP53 in high-grade serous ovarian tumor samples from the Cancer Genome Atlas project. Somatic mutations in TP53 was queried from cBioPortal. The query included 398 patient samples and 374 mutations were documented. Among them, 18 mutations are observed at R248 and R273 positions and 16 mutations are observed at R175. For R273 hotspot mutations, 9 R273H, 5 R273C, 3 R273L, and 1 R273P are reported. For R248 hotspot mutations, 10 R248Q and 8 R248W are reported. For R175 mutations, 15 R175H and 1 R175G are reported.

Figure S2

A

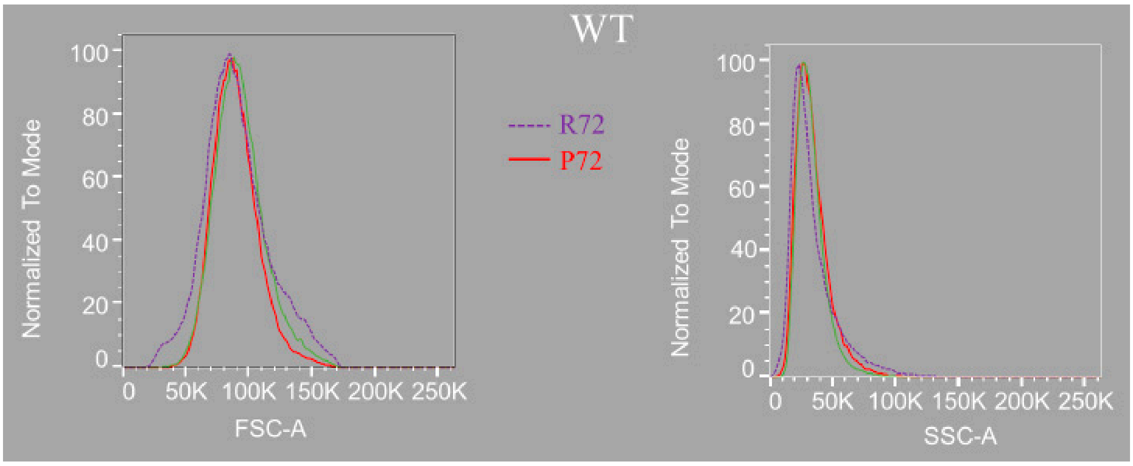
WT
R72
P72
Normalized To Mode
FSC-A
SSC-A
0
50K
100K
150K
200K
250K
20
40
60
80
100

B

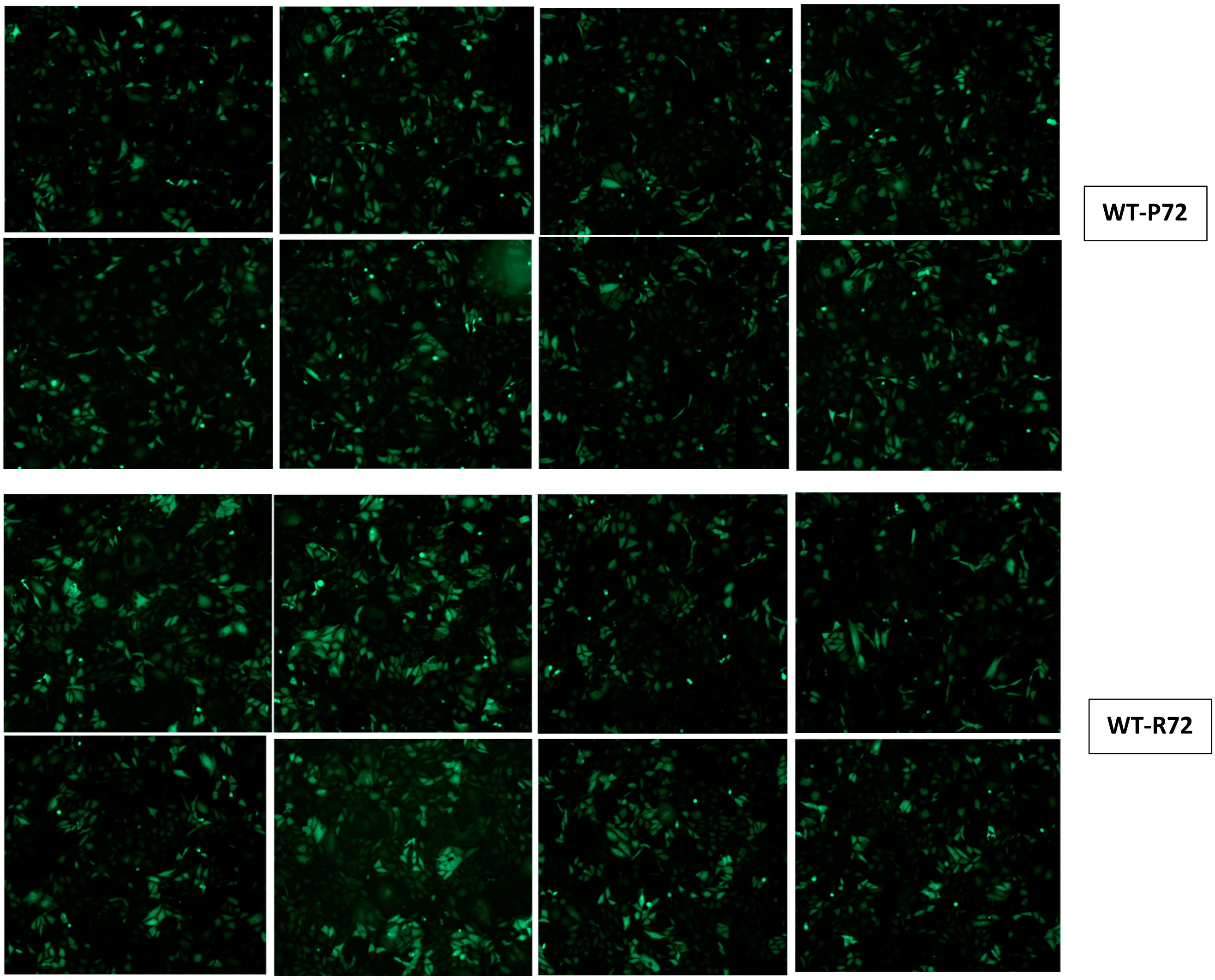
WT-P72
WT-R72

C

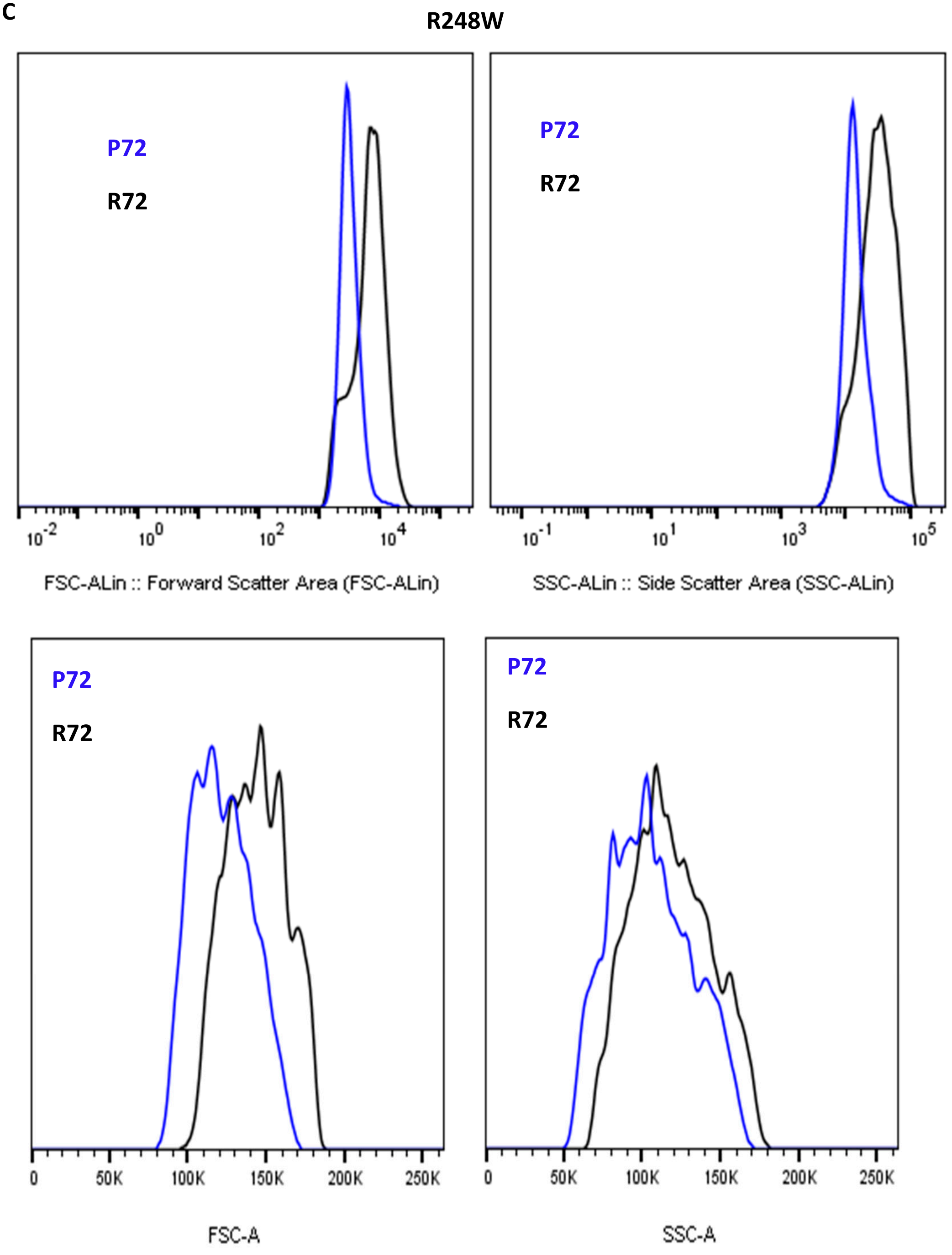
R248W
P72
R72
10^{-2} 10^{0} 10^{2} 10^{4}
FSC-ALin :: Forward Scatter Area (FSC-ALin)
P72
R72
10^{-1} 10^{1} 10^{3} 10^{5}
SSC-ALin :: Side Scatter Area (SSC-ALin)
P72
R72
0 50K 100K 150K 200K 250K
FSC-A
P72
R72
0 50K 100K 150K 200K 250K
SSC-A

D

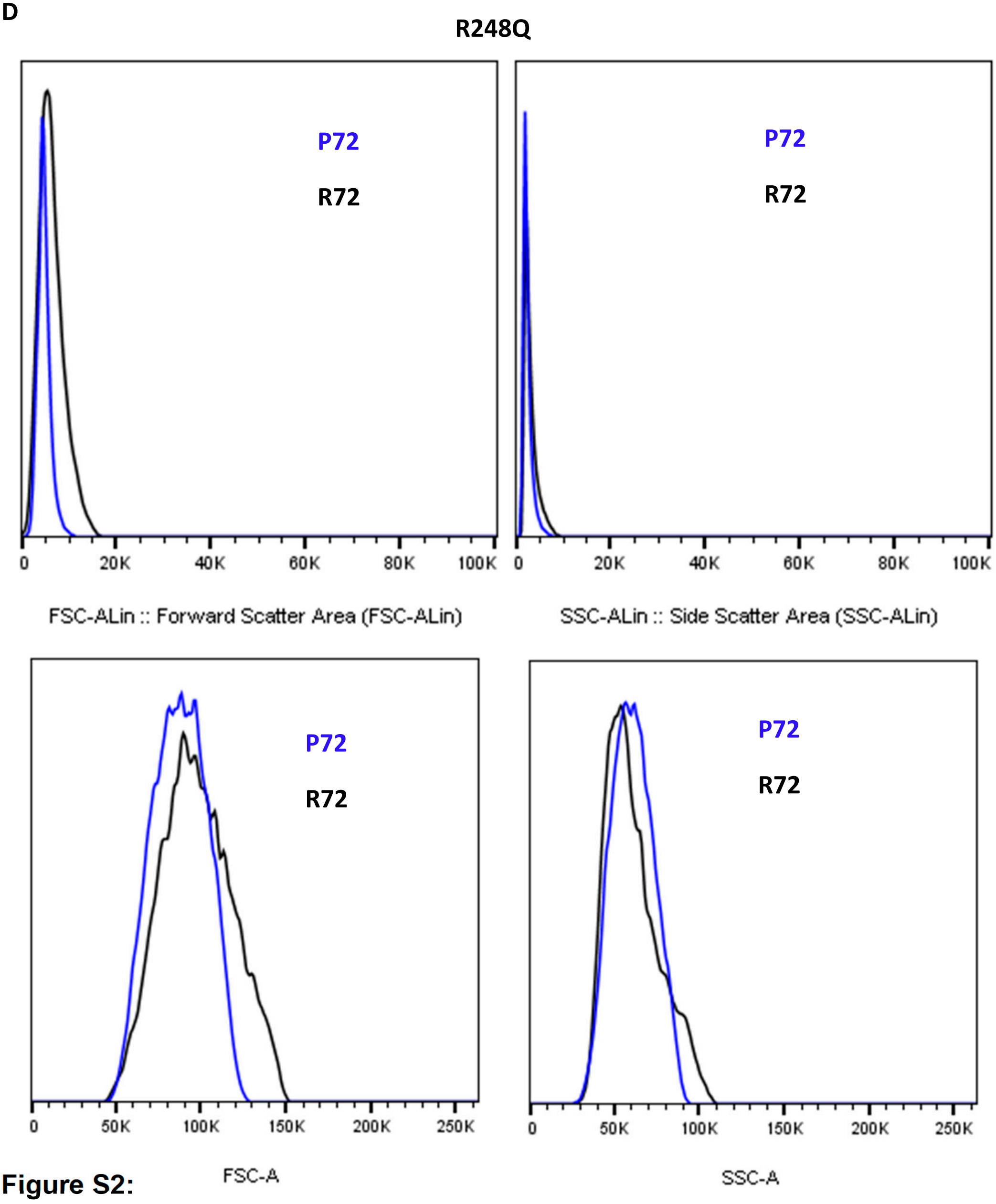

Figure S2:

The P72R SNP does not alter the morphology of wild type p53 cells. (A) Flow cytometry analysis showing the Forward Scatter (FSC) and Side Scatter (SSC) of wild type p53 with the P72 SNP (Red) and wild type p53 with the R72 (purple). SKOV3 parental cells (Green) were used as unstained control for gating. The mean FSC area of at least three independent clones that were pooled together was used. (B) Fluorescent microscopy images showing the morphology of wild type p53 with the P72 vs R72 are shown. FSC Chi-Squared T(X) = 0.83 and SSC Chi-Squared T(X) = 1.93. (C) Replicates of flow cytometry analyses of the FSC and SSC of R248W mutants. FSC and SSC Chi-Squared T(X) > 4. (D) Replicates of flow cytometry analyses of the FSC and SSC of R248Q mutants. FSC and SSC Chi T(X) > 4.

Figure S3

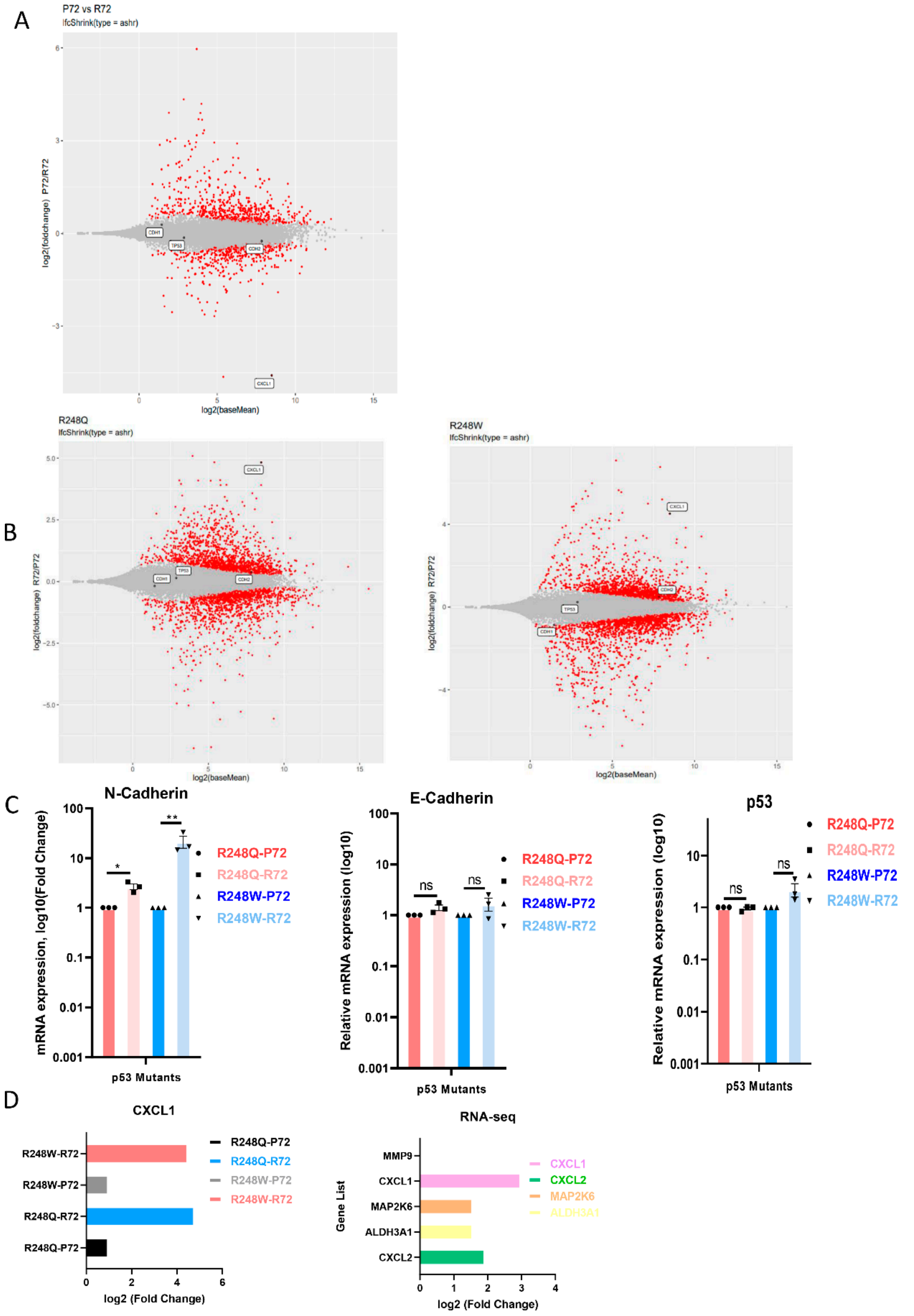

A
P72 vs R72
lfcShrink(type = ashr)
log2(foldchange) P72/R72
log2(baseMean)
CDH1
TP53
CDH2
CXCL1
B
R248Q
lfcShrink(type = ashr)
log2(foldchange) R72/P72
log2(baseMean)
R248W
lfcShrink(type = ashr)
C
N-Cadherin
mRNA expression, log10(Fold Change)
p53 Mutants
E-Cadherin
Relative mRNA expression (log10)
p53
R248Q-P72
R248Q-R72
R248W-P72
R248W-R72
ns
D
CXCL1
log2 (Fold Change)
RNA-seq
Gene List
MMP9
CXCL1
MAP2K6
ALDH3A1
CXCL2

E

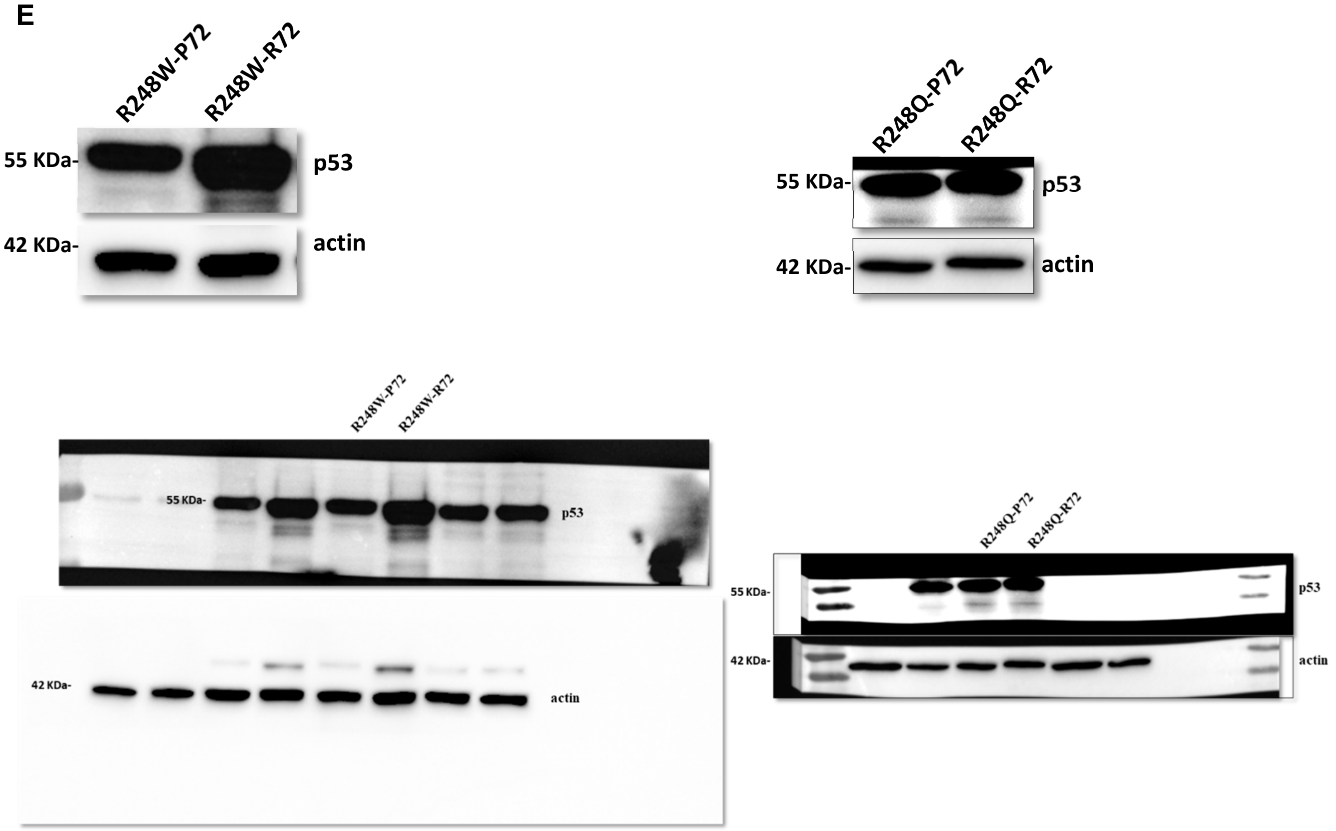

Figure S3: (A) MA plots showing *CXCL1* is significantly upregulated in both mutants (R248Q and R248W) with the R72 SNP. (B) MA plots showing the P72R SNP alters the invasion profile of mutant p53 via CXCL1 in each mutant R248W and R248Q. Data are displayed as Log2 Fold Change. (C) RNA expression profile between the two mutant pairs (R248W and R248Q) for EMT markers N-cadherin, E-cadherin and for p53. data shown as mean ± SEM. The statistical analysis was performed using two-tailed Student’s t-test: *p≤0.05, **p≤0.01, ***p≤0.001, ****p≤0.0001.(D) Graphs showing significant upregulation of CXCL1 between p53 mutants R248W and R248Q. (D) Additional transcripts significantly altered between the two groups (mutants + P72 SNP and mutants + R72 SNP) are shown (*ALDH3A, MAP2K6, CXCL2*). (E) Western blots showing p53 expression in R248W P72 vs R72 and R248Q P72 vs R72.

Figure S4

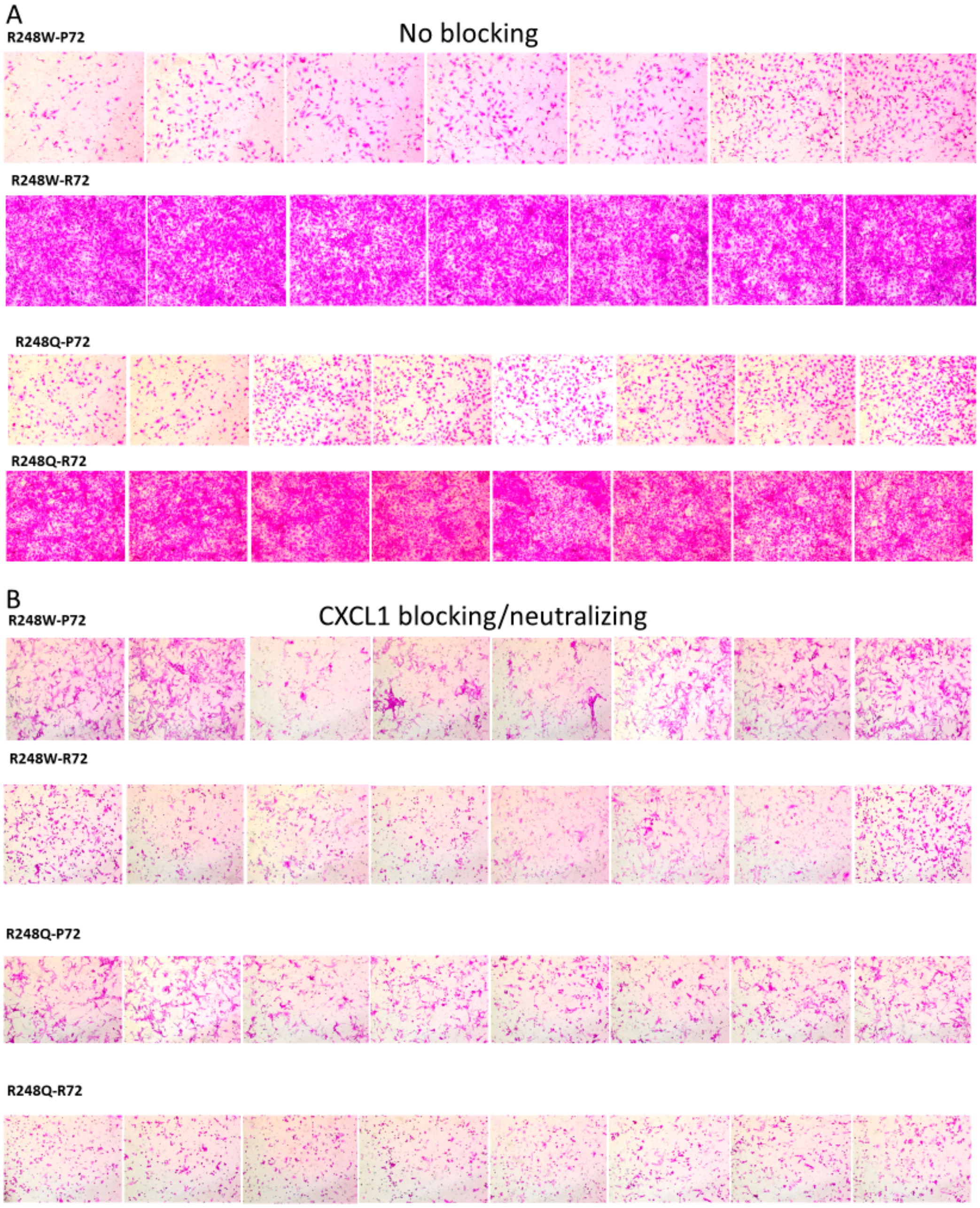

Figure S4: (A) All images representative of three independent experiments show the differential invasion profile of SKOV3 cells with conditioned media from p53 mutants R248W P72 versus R248W R72 and R248Q P72 versus R248Q R72. (B) All images representative of three independent experiments show the differential invasion profile of SKOV3 cells with conditioned media from p53 mutants R248W P72 versus R248W R72 and R248Q P72 versus R248Q R72 after the conditioned media is blocked with CXCL1 neutralizing antibody.

Figure S5

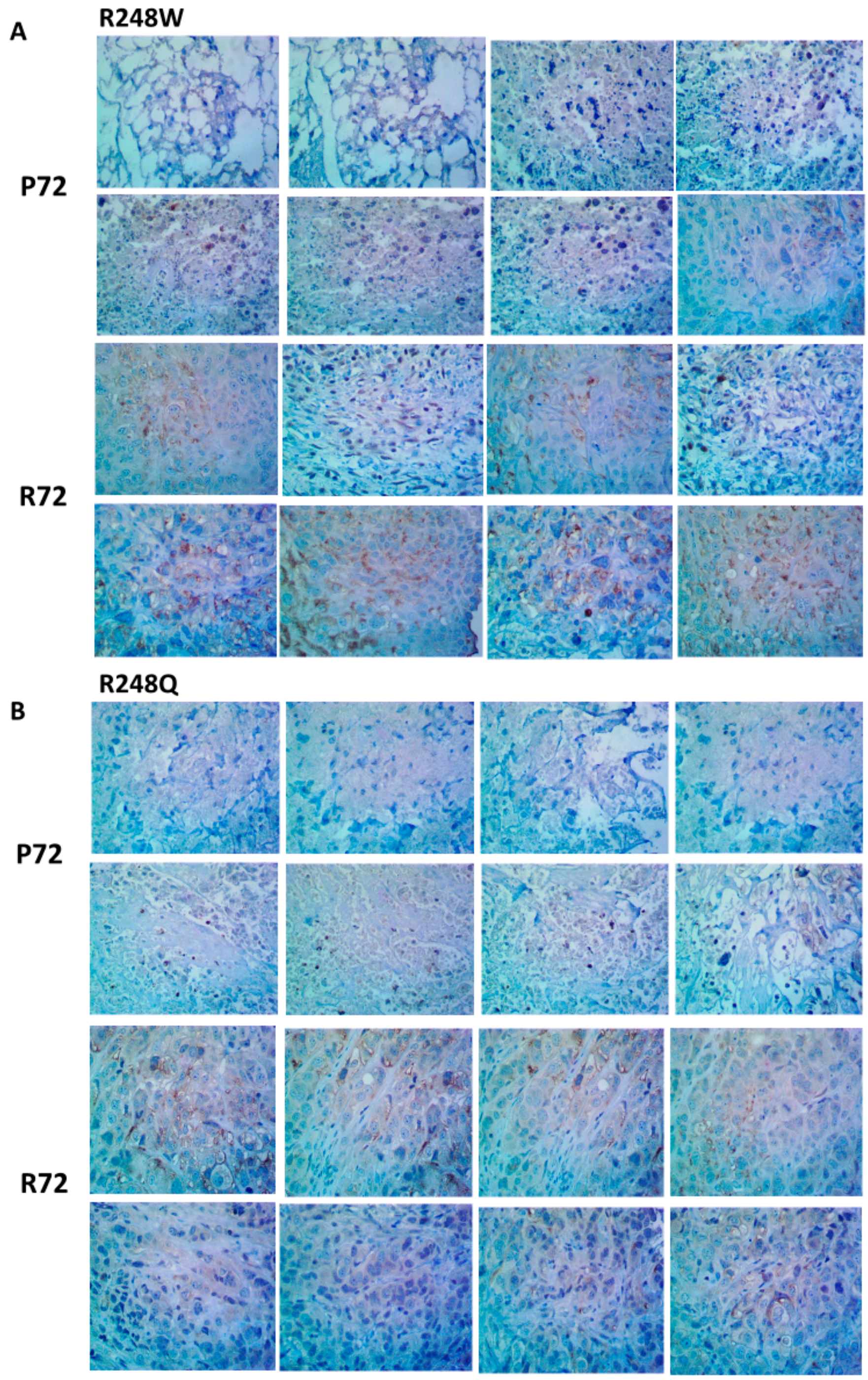

Figure S5: All images representative of tissue from independent tumor samples. (A) Immunohistochemistry staining of CXCL1 in mutant R248W P72 versus R248W R72. (B) Immunohistochemistry staining of CXCL1 in mutant R248Q P72 versus R248Q R72.

Figure S6

A

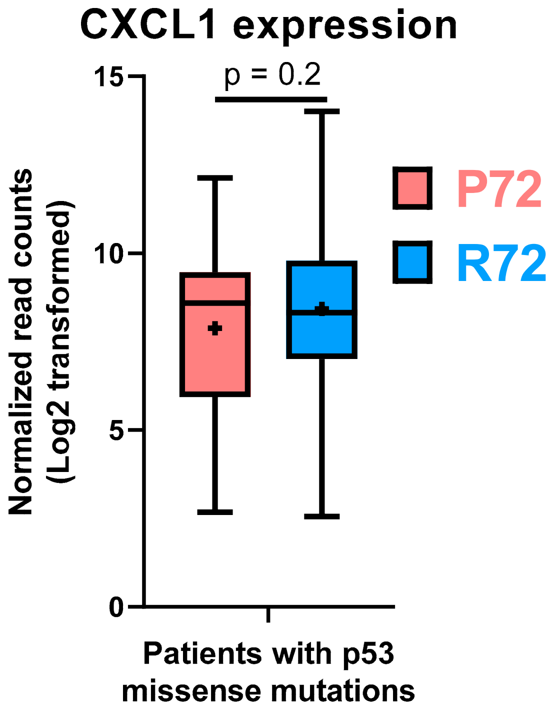

B

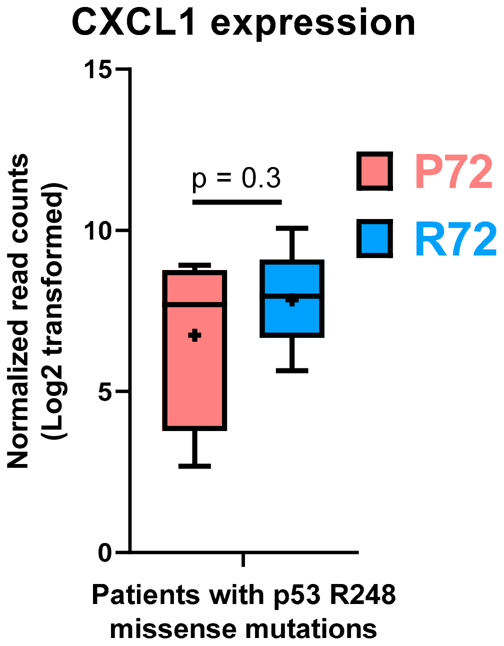

Figure S6: (A) Log 2-Fold Change indicating gene expression of CXCL1 in patients with all types of p53 mutations in the background of the P72 or R72 SNP. (B) Log 2-Fold Change indicating gene expression of CXCL1 in patients with R248 p53 mutations in the background of the P72 or R72 SNP.

Figure S7

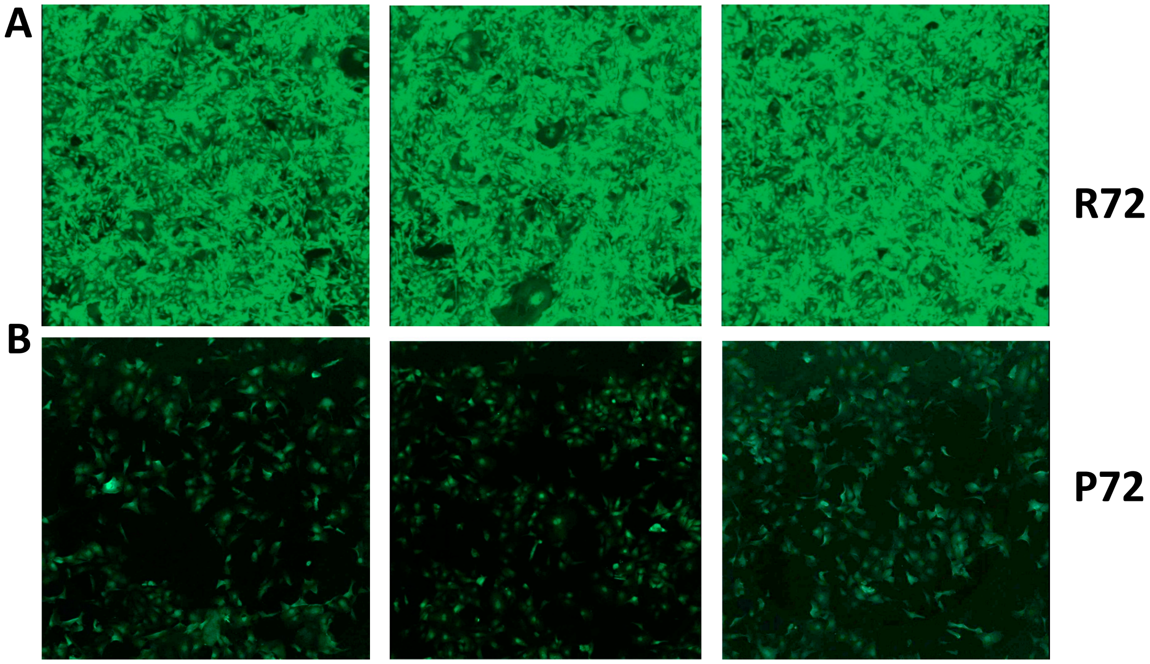

Figure S7: (A-B) GFP fluorescence images indicating higher expression of GFP in the cells transduced cells with the R248W mutants with the R72 polymorphism compared to the corresponding mutants with the P72 polymorphism. Cells were transduced for 5-7 days.

Table S1: Primer sequences used for RT-qPCR experiments.

CDH1 (Forward)	CATCGCTTACACCATCCTCAG
CDH1 (Reverse)	ACTCCTGTGTTCCTGTTAATGG
CDH2 (Forward)	CCCAAGACAAAGAGACCCAG
CDH2 (Reverse)	GCCACTGTGCTTACTGAATTG
CXCL1 (Forward)	CATCCAAAGTGTGAACGTGAAG
CXCL1 (Reverse)	GTCACTGTTCAGCATCTTTTCG
